# Untargeted plasma metabolomics reveals systemic metabolic dysregulation and reatment-associated metabolic modulation following YWKS treatment in sleep disorder patients

**DOI:** 10.3389/fphar.2026.1905102

**Published:** 2026-09-11

**Authors:** Anwar Abdurahman, Ailiyaer Yasheng, Aikelidan Abulajiang, Yang Liu, Xuehai Ma, Chenke Zhu, Ziqi Su, Jie Xu, Jiaying Chen, Wudi Kou, Ningjing Ge, Xieriye Mamutijiang, Dilinaerayi Yusupu, Jin Chen, Aizezi Aihemaitiniyazi

**Affiliations:** 1 Xinjiang Key Laboratory of Mental Development and Learning Science, School of Psychology, Xinjiang Normal University, Urumqi, Xinjiang, China; 2 The Second People’s Hospital of Xinjiang Uyghur Autonomous Region, Key Laboratory of Evidence-Based and Translation, Xinjiang Hospital preparation of Traditional Chinese Medicine, Urumqi, Xinjiang, China

**Keywords:** LC-MS/MS, metabolomics, sleep disorder, untargeted metabolomics, Yinao Wusiti Kudusi syrup (YWKS)

## Abstract

Sleep disorders are common and significantly impact metabolic regulation, but the overarching blood metabolomic characteristics and how drugs for these disorders affect metabolism have not been adequately described. Blood plasma from three distinct cohorts-healthy individuals, sleep disorder patients (SDP), and those undergoing YWKS pharmacological therapy (SDPM)-was analyzed using untargeted metabolomics (LC-MS/MS). Following quality control, 352 metabolites representing 21 chemical groups were kept, and a combination of differential analysis, multi-omics enrichment, and correlation networks was used to describe metabolic changes and how they reacted to treatment. In contrast to the control group, in patients with SDP, 173 metabolites showed significant dysregulation (121 up, 52 down). Evidence from this initial population indicated that YWKS therapy was correlated with the treatment-associated modulation of 119 of the 352 identified metabolic markers, while 54 remained in a state of imbalance, suggesting that therapeutic coverage was only partially achieved in this restricted sample size. Our study identified five putative metabolic axes encompassing purine degradation and oxidative stress, imbalances in the tryptophan-serotonin circuit, compromised mitochondrial fatty acid β-oxidation, the nexus of glycerophospholipid restructuring and neuroinflammation, and the induction of the renin-angiotensin system. These associations provide a hypothetical framework for understanding metabolic dysregulation in sleep disorders and warrant further mechanistic investigation. Through KEGG enrichment analysis, several vital metabolic routes were underscored, notably those involving glycerophospholipids, arachidonic acid signaling, and nucleotides. These findings suggest that sleep disturbances are associated with a complex metabolic imprint within the bloodstream that is not fully resolved by YWKS therapy. The continued presence of long-chain acylcarnitines in the SDPM group indicates ongoing mitochondrial dysfunction, suggesting that mitochondrial-targeted strategies warrant further exploratory investigation as potential adjuncts in the management of sleep disorders.

## 1 Introduction

Sleep disorders are a common clinical syndrome characterized by decreased sleep quality, with physiological hyperarousal as the core pathological feature. Symptoms include difficulty falling asleep or maintaining sleep (Fernandez-Mendoza, 2025). Chronic insomnia disorder (CID) is the most frequent subtype and the central focus of this paper, identified by challenges in starting or continuing sleep for three nights or more each week, lasting for at least 3 months. Recent studies have shown that insomnia, especially in those with objective short sleep, significantly increases the risk of developing hypertension, type 2 diabetes, and Alzheimer’s disease (Ungvari et al., 2025). Without intervention, insomnia can continuously damage cardiovascular and cognitive functions by activating the hypothalamic-pituitary-adrenal axis and inducing systemic inflammatory responses (Abou Daya et al., 2025).

Yinao Wusiti Kudusi Syrup (YWKS, New Drug Preparation License No.M20041577) is a unique traditional Chinese medicine (TCM) blend comprising several key components such as lavender, Adiantum capillus-veneris L., Paeoniae Radix Rubra, Anchusa italica Retz, Glycyrrhiza uralensis Fisch, Foeniculum vulgare Mill, Celery Seeds, Althaea rose, Hyoscyamus niger L., Ficus concinna Miq, seedless raisin, Cordia dicholoma Forst.f, and sucrose. Lavender is one of the important medicinal materials in this preparation. It has pharmacological effects such as sedation, anti-anxiety, and sleep improvement (Wang et al., 2025; Can et al., 2024; Jiang et al., 2025). Its main active ingredients, such as linalool and linalyl acetate, are believed to exert their effects by regulating the excitability of the central nervous system and affecting neurotransmitter transmission (Hareng et al., 2024). YWKS is commonly used clinically to treat insomnia, sleep disorders, and other related conditions. However, there is still a lack of metabolomics research on this drug, especially its key ingredient, lavender, and related reports are limited. Therefore, it is necessary to conduct further research to elucidate its potential biological activity, metabolic changes, and mechanisms of action (Can et al., 2024; Hareng et al., 2024).

Metabolomics, through the systematic analysis of endogenous small molecule metabolites, can directly reflect the final functional output and dynamic homeostasis of the body under the combined effects of factors such as genes, environment (Liu et al., 2022), behavior (Wu et al., 2025), and sleep (Hou et al., 2022; Zhang R. H. et al., 2025). It provides a powerful tool for revealing biomarkers and pathological mechanisms of complex diseases. Recent metabolomics studies have begun to reveal the association between sleep duration, sleep quality, and alterations in specific metabolic pathways. Studies have found that sleep deprivation or insomnia is associated with shifts in the tryptophan-kynurenine metabolic pathway, changes in bile acid profiles, fatty acid metabolism disorders, and fluctuations in the levels of gut microbiota-related metabolites (Shorer et al., 2024; Lingaraju et al., 2024). These findings suggest that sleep disorders are accompanied by extensive metabolic reprogramming. However, most existing studies are based on sleep duration variability in healthy individuals or case-control designs. For patients with a confirmed diagnosis of chronic insomnia disorder, the specific plasma metabolic profile has not yet been clearly defined. More importantly, there is a lack of research that systematically assesses the remodeling effect of an intervention on these disordered metabolic networks. This dynamic comparison of disease state - post-intervention state is of key value for elucidating treatment mechanisms and discovering biomarkers of efficacy.

Based on this, this study aims to use non-targeted metabolomics technology to conduct a systematic analysis of three groups of people: patients with chronic insomnia disorder, healthy controls, and insomnia patients who have undergone standardized drug treatment. The core objective of this study is: first, to map and identify specific plasma metabolic profiles that distinguish chronic insomnia disorder from a healthy state. Second, assess the reversal and corrective effects of drug therapy on the aforementioned abnormal metabolic characteristics, thereby providing new insights into the treatment mechanism at the systemic metabolic level. This study is expected to provide new evidence for the biological basis of sleep disorders and lay the foundation for the development of objective assessment tools based on metabolites.

## 2 Materials and methods

### 2.1 Sample collection

Yinao Wusitikudus Syrup (YWKS) is an in-house preparation of Xinjiang Uygur Autonomous Region Uygur Medicine Hospital. It has been filed and approved by the provincial drug supervision and management department (approval number: M20041577) and is produced by the hospital’s preparation room for exclusive clinical use in the hospital’s specialties. The preparation is mainly composed of lavender, Adiantum capillus-veneris L., Paeoniae Radix Rubra, Anchusa italica Retz, Glycyrrhiza uralensis Fisch, Foeniculum vulgare Mill, Celery Seeds, Althaea rose, Hyoscyamus niger L., Ficus concinna Miq, seedless raisin, Cordia dicholoma Forst.f, and sucrose. Patients in the treatment group took YWKS syrup orally, 30 mL each time, twice daily, for 14 consecutive days.

This study included 21 human plasma samples, divided into three independent groups: a healthy control group (SD), a sleep disorder group (SDP), and a YWKS treatment group (SDPM), with seven patients in each group. SDP in both groups were diagnosed according to the ICSD-3 criteria (Please see the Supplementary Material sample collection section for details). Baseline Clinical Characteristics Please see the Supplementary Table S1.

### 2.2 Metabolite extraction and detection

After the protein was extracted from the sample by methanol precipitation, the supernatant was analyzed by non-targeted metabolomics detection in positive and negative ion mode using LC-MS/MS. The raw data were imported into Compound Discoverer 3.2 software to complete the extraction and alignment of characteristic peaks and the identification of metabolites based on secondary mass spectrometry (MS/MS).

### 2.3 Data preprocessing

Before multivariate statistical analysis, the raw metabolomics data were systematically preprocessed. Missing values were filled using a 1/5 minimum value strategy, followed by Pareto standardization and logarithmic transformation to eliminate the interference of extreme values of high-abundance metabolites and improve the data distribution.Based on the preprocessed data, an orthogonal partial least squares discriminant analysis (OPLS-DA) model was constructed using SIMCA 16 software to assess the differences in metabolomics characteristics among groups. To verify the robustness of the model and rule out overfitting, 200 permutation tests were performed on all pairwise comparisons (n = 200, Supplementary Figure S1). All models showed statistically significant Q^2^ values after permutation (p < 0.05), and the Q^2^ distribution after permutation was concentrated near zero, confirming that the separation results between groups were true and reliable, and that the models were not overfitting.

All 352 analyzed metabolites were cross-referenced with DrugBank and HMDB drug metabolite lists to minimize biases caused by exogenous substances, with compounds known or thought to originate from drugs tagged before initiating pathway or network analysis. These marked metabolites were kept for openness but left out of pathway and KEGG enrichment investigations, while their role in the correlation network is cited as a limitation in Section 3.6 to ensure that the five mechanistic axes in Section 3.5 focus on endogenous systems rather than pharmacokinetic interference.

### 2.4 Differential metabolite analysis

Student’s t-test was used to analyze differences in metabolites between groups, and FDR correction was applied. |log2FC| ≥ 0.3 (i.e., FC ≥ 1.23 or FC ≤ 0.81), FDR ≤0.05 and VIP ≥1.0 were used as the criteria for identifying differentially accumulated metabolites (DAMs).

### 2.5 Functional enrichment analysis

MetaboAnalyst 5.0 was used to perform KEGG pathway enrichment analysis and MSEA enrichment analysis on differential metabolites. KEGG enrichment was performed based on the hypergeometric test.

### 2.6 Metabolite-related network analysis

Spearman correlation analysis was used to assess the correlation between metabolites, and correlation pairs with |r|≥0.7 and p < 0.05 were retained to construct network edges. Subsequently, community detection was performed based on the Louvain algorithm of igraph, and visualization was performed using Cytoscape.

### 2.7 Statistical analysis

Statistical analysis was performed using R 4.3.0 and Python 3.11. Statistical significance across multiple groups was determined via one-way ANOVA, while pairwise comparisons were conducted using two-tailed Student’s t-tests adjusted for the false discovery rate (FDR). In two-tailed tests, *p* < 0.05 was considered statistically significant.

## 3 Results

### 3.1 Overview of data quality and metabolite coverage

**FIGURE 1:**
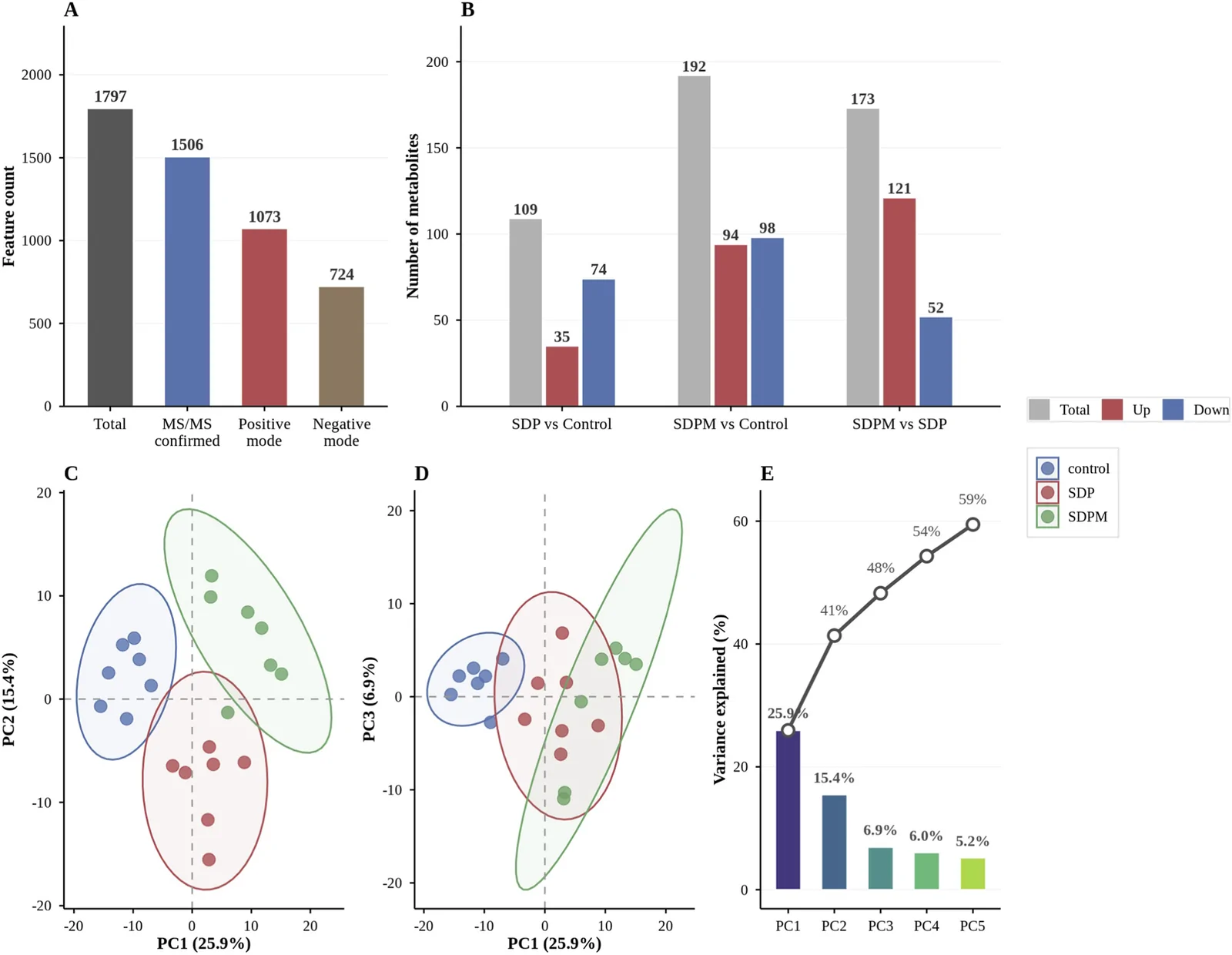
Results of quality control evaluation, metabolomics characterization, and principal component analysis. **(A)** Metabolite identification summary across ionization modes. **(B)** Differentially abundant metabolites (DAMs) for each pairwise comparison (|log_2_FC| > 0.3, *p* < 0.05, VIP ≥ 1.0). **(C–D)** PCA score plots; ellipses indicate 95% confidence regions. **(E)** Variance explained by the first five principal components; line and points show cumulative variance. The study included three groups of seven subjects each (Control A1–A7, SDP B1–B7, SDPM C1–C7; n = 21 total).

As illustrated in Figure 1A, 1797 metabolite features were found using both positive (ESI^+^)and negative (ESI^−^) ionization modes; among these, 1506 were tentatively identified based on MS/MS spectral matching. A total of 352 metabolites, spanning 21 chemical categories, were retained for subsequent evaluation after quality screening based on an RSD threshold of ≤30% in QC samples. The primary chemical groups identified were benzene and substituted derivatives (n = 63,17.9%), along with fatty acids (n = 42, 11.9%) and organic acids (n = 41, 11.6%). The instrument remained stable throughout the analytical run, demonstrated by high reproducibility and a median RSD of 6.8% in QC samples. Significant metabolic differences between the experimental groups were identified through pairwise differential abundance testing, as shown in Figure 1B. In comparison to the healthy control cohort, subjects with SDP displayed 173 metabolites of differential abundance (121 upregulated and 52 downregulated), a finding backed by a strong OPLS-DA model (R^2^Y = 0.996, Q^2^ = 0.578; Supplementary Table S2). In contrast to controls, 192 DAM were identified in the SDPM group (98 upregulated and 94 downregulated), supported by a correspondingly validated OPLS-DA model (R^2^Y = 0.996, Q^2^ = 0.68; Supplementary Table S3). A higher frequency of DAMs in the SDPM group (192 vs. 173) and a balanced parity between upregulated 94 and downregulated 98 metabolites suggest that the treatment elicited supplementary metabolic modifications beyond pathological mitigation; this phenomenon reflects drug-specific metabolic restructuring as opposed to a failure to achieve the healthy homeostatic baseline. The application of unsupervised PCA resulted in the formation of three distinct, non-overlapping groups in the PC1-PC2 and PC1-PC3 score plots, with 95% confidence ellipses used to define the boundaries of each group as seen in Figures 1C,D. These results offer separate validation for unique metabolic profiles linked to the stage of the disease and how well the treatment works. PC1 and PC2 explained 25.9% and 15.4% of the overall variation, while PC3 added another 6.9% (Figure 1E).

Collectively, about 60% of the cumulative variance was explained by the initial five principal components, highlighting the inherent high-dimensional complexity found in the plasma metabolome.

### 3.2 Differential metabolite analysis

Comparison of SDP patients with healthy controls identified 173 significantly differentially abundant metabolites (DAMs); |log_2_FC| > 0.3, P < 0.05, VIP ≥1.0). The OPLS-DA model demonstrated robust separation (R^2^Y = 0.996, Q^2^ = 0.578), indicating a highly discriminative metabolic phenotype (Supplementary Table S2).

**FIGURE 2:**
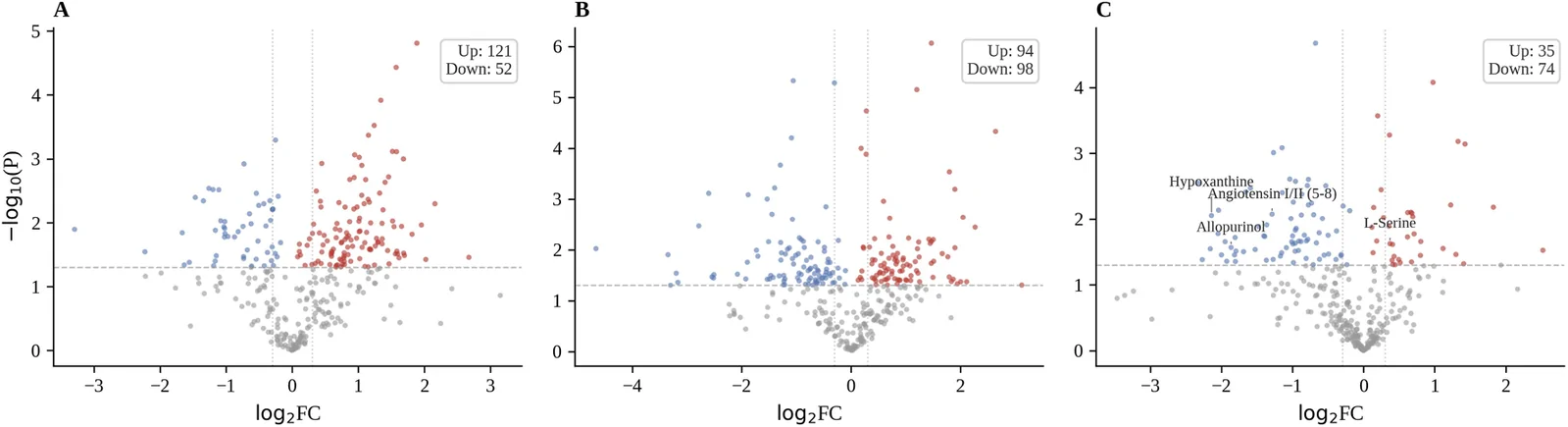
Volcano plots showing differentially abundant metabolites (DAMs) across the three pairwise comparisons. Thresholds: |log_2_FC| > 0.3, P < 0.05, VIP ≥1.0. Red: upregulated; blue: downregulated; grey: not significant. **(A)** Contrast of SDP and Control resulted in 173 DAM (121 up, 52 down). **(B)** Comparing SDPM/Control found 192 DAM (94 up, 98 down). **(C)** Contrast of SDPM and SDP found 109 DAM (74 drug-lowered, 35 drug-raised), featuring annotations for key metabolites.

**FIGURE 3:**
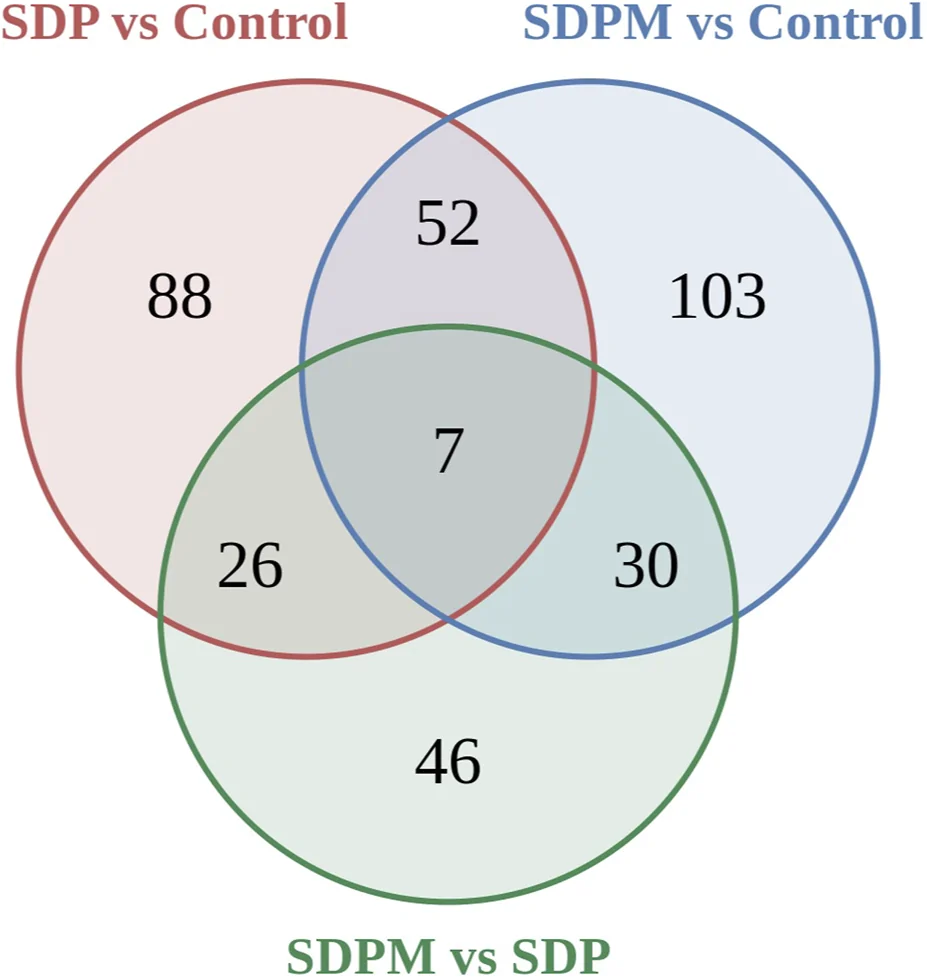
Venn diagram analysis of three groups of differentially metabolites.

Among the metabolites showing significant upregulation were hypoxanthine (log_2_FC = +1.96), stearoylcarnitine (+2.16), 5-hydroxyindole (+1.58), and neuroendocrine indicators such as estriol (+1.36) and allotetrahydrocorticosterone (+1.47), whereas there was a notable reduction in arachidonic acid-containing glycerophospholipids, specifically PC(16:0/20:4) (−1.26) and PE (18:0/20:4) (−0.82) (Figure 2A). As shown in (Figure 2B), the SDPM group still displayed 192 DAM when compared with controls despite receiving pharmacological treatment, evidencing partial metabolome restoration. Comparing SDPM and SDP directly showed 109 DAM associated with theYWKS treatment, 74 of which were reduced and 35 elevated, with hypoxanthine, hippuric acid, and angiotensin I/II (5–8) exhibiting the strongest reversals; notably, stearoylcarnitine stayed persistently high compared to controls, indicating a therapeutic gap in mitochondrial-oxidation (Figure 2C). As shown by the Venn diagram, the three pairwise comparisons exhibited only partial sharing of DAMs, which denotes that YWKS treatment resulted in an incomplete modulation of metabolic processes (Figure 3). A total of 25 DAM were found to overlap in the SDPM comparisons, with 21 overlapping in the SDP-related ones, and a group of seven metabolites common to all three comparisons formed a central pharmacologically modulated profile of sleep disorders.

### 3.3 Response pattern classification

For a total overview of metabolic behavior in the three groups, all 352 detected metabolites were grouped into seven response styles based on their direction in SDP vs. Control and SDPM vs. SDP comparisons (Supplementary Table S3). YWKS treatment was associated with partial normalization of 119 of the 173 disease-linked DAMs (68.8%), whereas 54 DAM (31.2%) continued to show dysregulation, highlighting a clear limitation in the therapy’s effectiveness, which indicates a measurable gap in treatment efficacy. A further 133 metabolites exhibited drug-specific alterations that were not directly correlated with disease-related disruptions.

**FIGURE 4:**
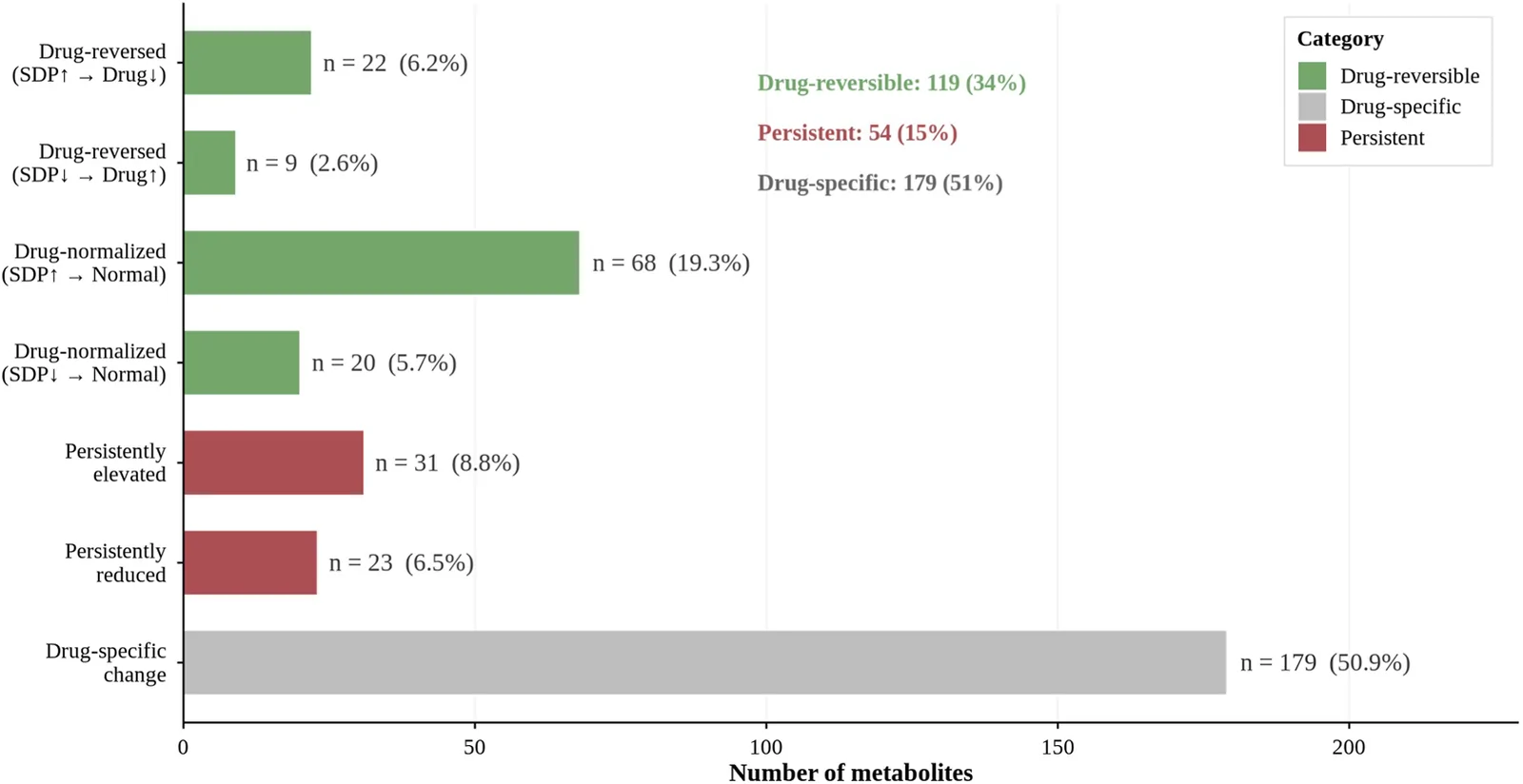
illustrates the classification of multi-group response patterns.

However, 54 persistently altered metabolites (categories 5–6; 15%) remained dysregulated regardless of treatment. Key persistently elevated metabolites included: stearoylcarnitine (FA class, log_2_FC_SDP = +2.16), nervonic acid (FA, log_2_FC = +0.39), triethylamine, and MG (18:1/0:0/0:0). The persistent elevation of multiple long-chain acylcarnitines suggests an unresolved mitochondrial β-oxidation defect (Figure 4).

**FIGURE 5:**
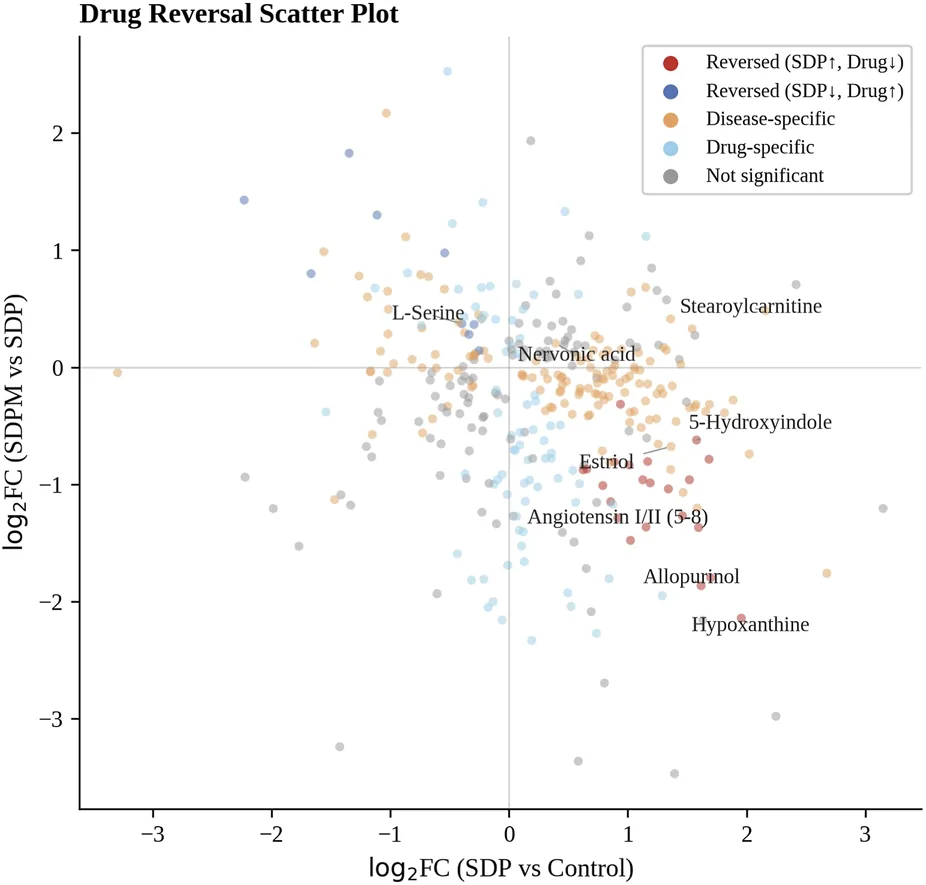
Displays a drug reversal scatter plot with the x-axis showing log_2_FC SDP relative to Control and the y-axis showing log_2_FC SDPM relative to SDP. Metabolites reversed by the medication are situated in quadrants II and IV. Notably identified substances include hypoxanthine, which displays the most significant reversal (log_2_FC = −2.14), stearoylcarnitine, which exhibits no reversal and indicates a therapeutic gap, angiotensin I/II (5–8) (log_2_FC = −1.29), and 5-hydroxyindole (log_2_FC = −0.62).

In order to compare pharmacological reversal against disease-state imbalances, a scatter plot (Figure 5) was developed using log_2_FC (SDP vs. Control) for the x-axis and log_2_FC (SDPM vs. SDP) for the y-axis. The metabolites that show drug-induced reversal are situated in the second and fourth quadrants. The most significant reversal was observed for hypoxanthine (log_2_FC_SDP = +1.96; log_2_FC_drug = −2.14), whereas stearoylcarnitine demonstrated no such effect (log_2_FC_drug = +2.64 compared to Control), reinforcing its role as a therapeutic gap metabolite through visual evidence. The significant reversal of Angiotensin I/II (5–8) (log_2_FC_drug = −1.29) and the limited reversal of 5-hydroxyindole (log_2_FC_drug = -0.62) support the hypothesis that YWKS modulates the serotonin and RAS pathways.

### 3.4 KEGG pathway enrichment and MSEA

**FIGURE 6:**
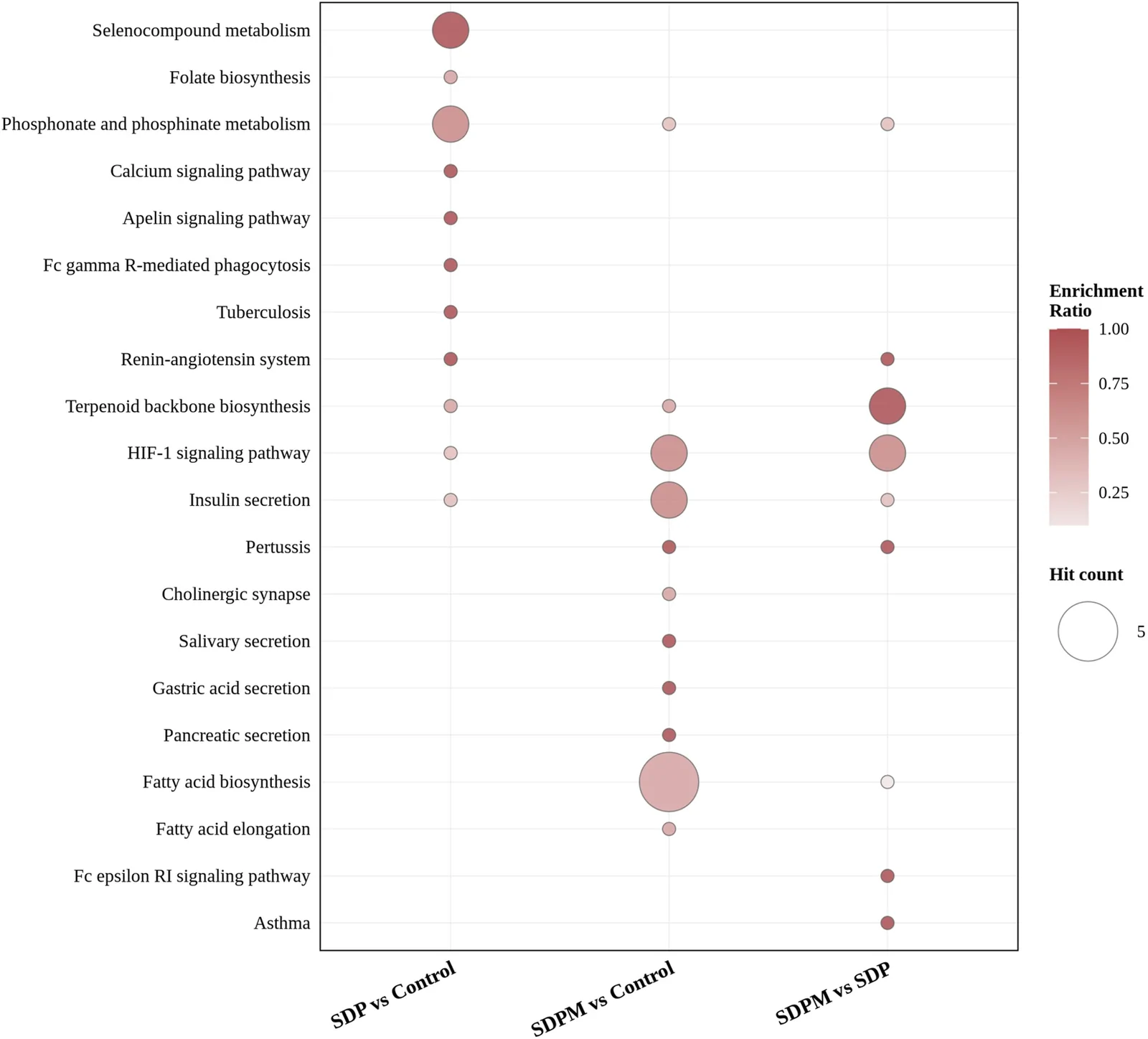
KEGG pathway enrichment across the three pairwise comparisons. The size of each bubble indicates how many differentially abundant metabolites are associated with a given pathway, with the color’s shade representing the enrichment ratio. To better showcase specific changes in metabolism, wider global pathways were not included.

As indicated in Supplementary Table S4, KEGG pathway enrichment analysis highlighted different pathway enrichments throughout all three comparison sets. Analysis of SDP against the Control showed that glycerophospholipid metabolism was the highest enriched pathway (9/81 metabolites, *p* < 0.05), while other notable pathways included cysteine and methionine metabolism (5/18), D-amino acid metabolism (5/30), ABC transporters, and the biosynthesis of cofactors. Comparing SDPM to the Control, enrichment trended toward lipid-associated pathways, with linoleic acid metabolism, arachidonic acid metabolism, and retrograde endocannabinoid signaling each recording at least 10 hits, *p* < 0.01, which aligns with the drug’s effect on inflammatory eicosanoid and endocannabinoid signaling. In the SDPM versus SDP comparison, glycerophospholipid metabolism was the primary drug-impacted pathway with seven hits, alongside carbon and glyoxylate/dicarboxylate metabolism. Significantly, the metabolic pathways of glycerophospholipids were enriched throughout all three comparisons, reinforcing their key role in the pathophysiology of sleep issues and pharmacological outcomes (Figure 6). Independent verification through Metabolite Set Enrichment Analysis (MSEA), conducted without pre-defined differential cutoffs, showed that the overall plasma metabolome in SDP was markedly enriched with metabolite groups related to lactic acidosis *(p* < 0.05), pyruvate metabolism *(p* < 0.01), and mitochondrial impairment *(p* < 0.001). The identified enriched clusters align with markers seen in epilepsy, Alzheimer’s, and other neurodegenerative conditions, situating sleep disorders in a more expansive context of metabolic and neurological diseases linked by mitochondrial and energy deficits.

### 3.5 Integrated multi-axis mechanistic analysis

As indicated in Supplementary Table S5, five mechanistic axes comprising purine breakdown, tryptophan–serotonin imbalance, impaired fatty acid oxidation, neuroinflammation, and renin–angiotensin activity were pinpointed to explain YWKS’s effectiveness by separating persistent biomarkers from successfully addressed targets.

**FIGURE 7:**
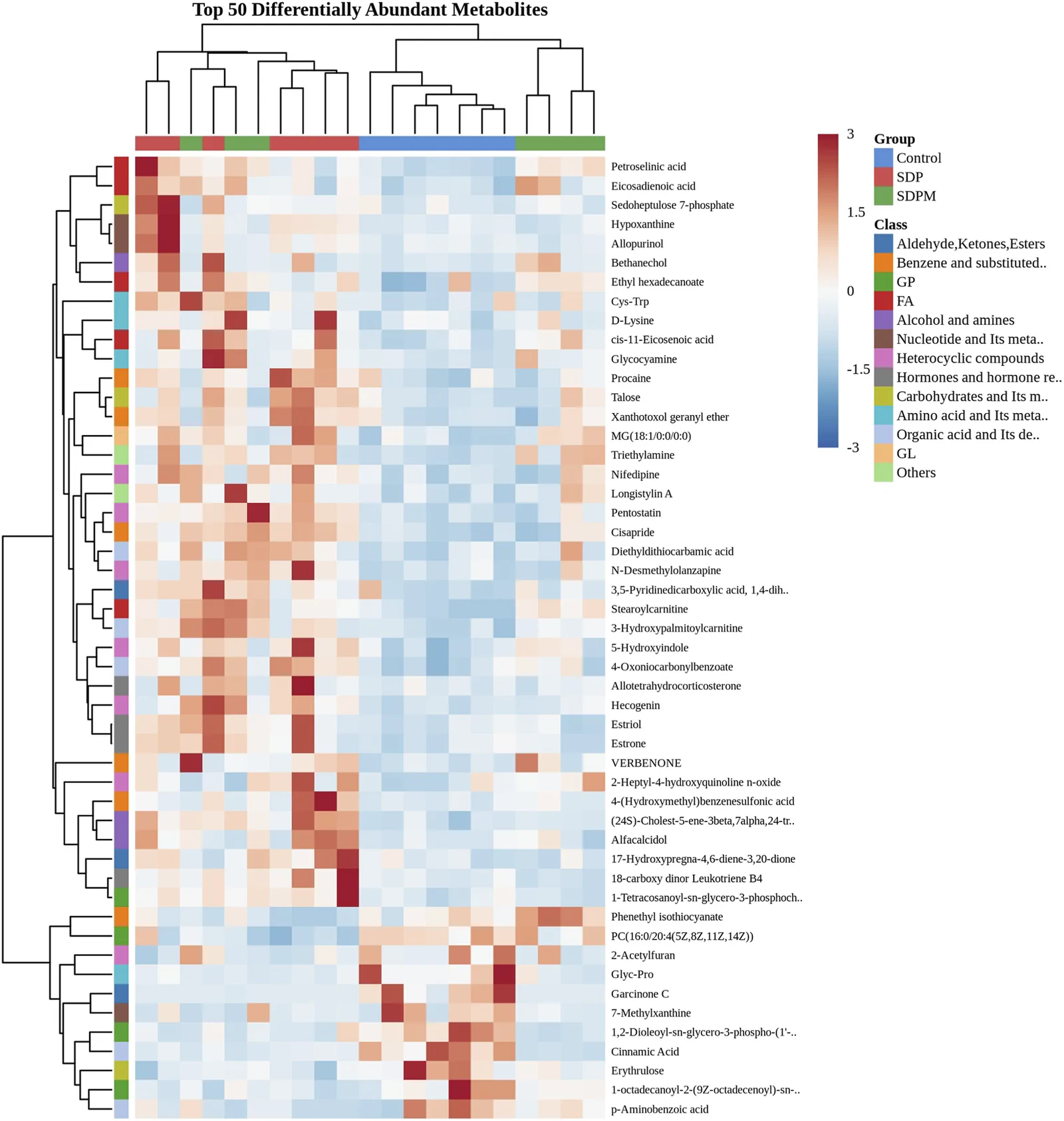
Heatmap of the top 50 differentially abundant metabolites (DAMs) across all 21 samples using z-score normalization. Columns are color-coded by group (Control, blue; SDP, red; SDPM, green), and row-side bars indicate metabolite chemical classes. Hierarchical clustering clearly separated the three groups, with SDP patients showing distinct metabolic dysregulation compared to controls, while the SDPM group exhibited partial metabolic restoration following YWKS treatment.

As shown in Figure 7, hierarchical clustering based on the top 50 differential metabolites clearly differentiated SDP patients from healthy controls, with the YWKS-treated (SDPM) group exhibiting a transitional profile that suggests a trend toward partial metabolic normalization.

**FIGURE 8:**
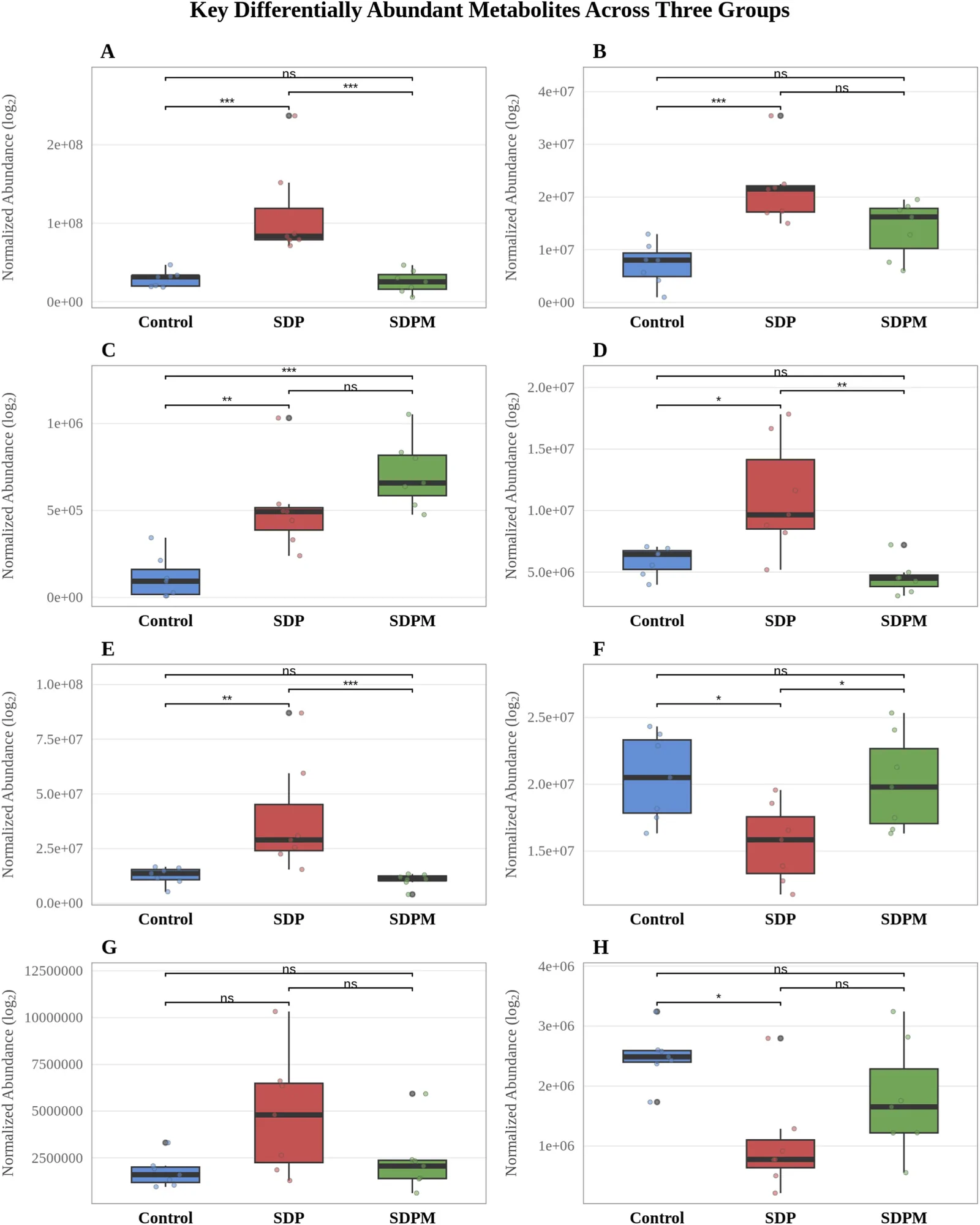
Box plots illustrating important DAMs for Control (blue), SDP (red), and SDPM (green) groups: **(A)** Hypoxanthine; **(B)** 5-hydroxyindole; **(C)** stearoylcarnitine; **(D)** allopurinol; **(E)** angiotensin I/II (5–8); **(F)** L-serine; **(G)** allotetrahydrocorticosterone; **(H)** PC(16:0/20:4).

Figure 7 A heatmap illustrates the hierarchical clustering of the top 50 DAM for all 21 samples using z-score normalization. The columns are color-coded by group (Control is blue, SDP is red, and SDPM is green), whereas the row-side bars identify chemical categories and response patterns. Box plots for eight key DAMs validated the group-specific trends (Figures 8A–H), showing that hypoxanthine and allopurinol increased together in SDP but were notably countered by YWKS (Figures 8A,D); The persistent elevation of stearoylcarnitine in SDPM is consistent with a mitochondrial axis that may be resistant to treatment (Figure 8C); As seen in Figure 8B, 5-hydroxyindole demonstrated only partial normalization; Figures 8E–H demonstrate that angiotensin I/II (5–8), L-serine, allotetrahydrocorticosterone, and PC(16:0/20:4) underwent substantial changes in SDP and showed trends toward partial normalization following therapy.

### 3.6 Correlation network analysis

**FIGURE 9:**
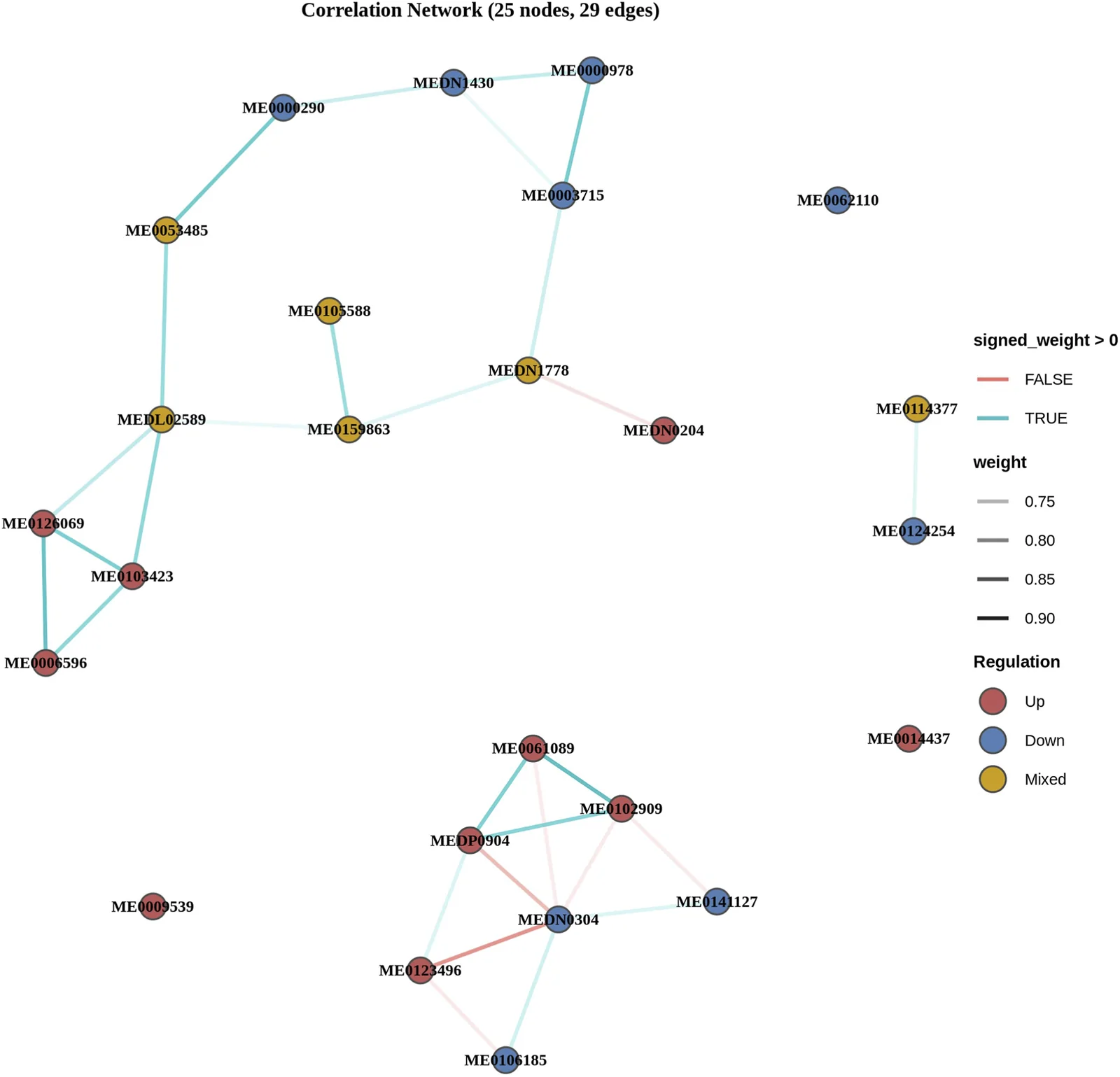
Metabolite correlation network: 25 hubs and 29 key edges. The full network comprised 352 nodes and 1137 significant pairwise correlations (|r| ≥ 0.7, *P* < 0.05), with an average node degree of 6.46. Subnetwork shows top-connected hub metabolites from 89 Louvain-detected modules. Node color marks chemical class; edge weight shows correlation strength.

Spearman correlation analysis of all 352 metabolites identified 1,137 significant pairwise correlations (|r| ≥ 0.7, P < 0.05). The resulting network had an average degree of 6.46 and contained 89 functional modules identified by Louvain community detection (Figure 9). Key hub metabolites (degree ≥30) included Mebhydrolin (degree = 40), Cisapride (degree = 40), and Metoclopramide (degree = 35). The top hub metabolites (Mebhydrolin, Cisapride, Metoclopramide) are exogenous pharmaceutical compounds flagged in Section 2.3; their high connectivity likely reflects co-administration patterns rather than endogenous regulation, and no mechanistic conclusions are drawn from their hub positions. Allopurinol is similarly interpreted with caution, as its levels may reflect medication use rather than endogenous purine metabolism alone.

These high-degree nodes predominantly belonged to the Benzene derivatives and Heterocyclic compounds classes, suggesting their central roles in orchestrating metabolic network responses to sleep disorder and pharmacological intervention.

## 4 Discussion

This study provides a systematic and comprehensive characterization of the plasma metabolome of patients with sleep disorders and, for the first time, compares the metabolic response characteristics before and after YWKS drug intervention in the same cohort. Based on differential analysis of 352 metabolites, seven-category response pattern classification, KEGG pathway enrichment, and correlation network analysis, we identified five putatively interconnected metabolic axes, providing associational evidence for multi-system metabolic dysregulation in sleep disorders and the boundaries of drug response.

Hypoxanthine was the metabolite with the largest increase in the SDP group (log_2_FC = +1.96), and it also had the most significant reversal effect of YWKS intervention (log_2_FC = −2.14)**.** As a core substrate of XO, its oxidative metabolism directly generates superoxide anions, which drive the accumulation of uric acid and purine intermediates, triggering oxidative stress and endothelial dysfunction (Ungvari et al., 2025; Fernandez-Mendoza, 2025), and its level is significantly negatively correlated with the sleep quality index (Zhang R. H. et al., 2025). The SDP group also showed elevated purine levels (log_2_FC = +1.62). Considering its exogenous XO inhibitor properties, this may be due to the medication background or ambiguity in the MS/MS identification of purine compounds, requiring further investigation. The synchronous reversal of both by YWKS (log_2_FC_drug = −1.86 for allopurinol) suggests its targeted regulation of the purine-XO axis. Furthermore, MSEA analysis showed that the SDP group was significantly enriched in the metabolites of lactic acidosis (*p* < 0.05) and pyruvate metabolism (*p* < 0.01), overlapping with the metabolic characteristics of neurodegenerative diseases, suggesting a potential association between sleep disorders and mitochondrial energy metabolism impairment, though causal directionality remains to be established (Zhang J. et al., 2025).

Five- Hydroxyindole was significantly upregulated in the SDP group (log_2_FC = +1.58). As a metabolic intermediate of MAO-catalyzed serotonin oxidation, its accumulation reflects excessive peripheral 5-HT degradation and decreased central 5-HT energy tone, impairing sleep-wake cycle homeostasis (Xu et al., 2025). Meanwhile, pathological shunting of the tryptophan-kynurenine pathway also intensifies neuroinflammation by competitively depleting 5-HT and melatonin precursors. (Kaluski-Kopatch et al., 2025; Etchegaray et al., 2013). YWKS intervention only partially reversed the effects (log_2_FC_drug = −0.62). Although its core component, linalool, can indirectly stabilize 5-HTergic signals via GABA_A receptors (Wang et al., 2025; Jiang et al., 2025; Chen et al., 2024), its direct inhibition of MAO-mediated degradation is still insufficient, suggesting that this pathway requires combined targeted intervention.

Stearoylcarnitine was significantly upregulated in the SDP group (log_2_FC = +2.16), and instead of decreasing after YWKS treatment, it increased (log_2_FC vs. Control = +2.64), making it the most prominent treatment gap metabolite in this study. The accumulation of long-chain acylcarnitine in plasma is a classic marker of mitochondrial β-oxidation impairment-chronic sleep deprivation can damage mitochondrial membrane potential via the HIF-1α pathway, leading to the inhibition of long-chain acyl-CoA oxidation and its entry into the blood as acylcarnitine (Ping et al., 2024; Kankuri et al., 2023; Song et al., 2023). MSEA also detected a highly significant enrichment signal in the set of mitochondrial damage-related metabolites (*p* < 0.001), supporting the presence of this persistent pathological feature. Meanwhile, the persistently elevated nervonic acid (log_2_FC = +0.39) suggests myelin metabolism disorder, which echoes reports of impaired white matter integrity in patients with chronic insomnia (Qu et al., 2026). The above findings collectively support the inclusion of mitochondrial-targeted adjuvant therapies (such as coenzyme Q10 and L-carnitine) in comprehensive intervention programs for sleep disorders (Mantle and Hargreaves, 2025; Bagheri et al., 2023).

Glycerol phospholipid metabolism was the only pathway that was consistently significantly enriched in pairwise comparisons among the three groups (SDP vs. Control: 9/81; SDPM vs. SDP: 7/81, both p < 0.05). The SDP group showed a significant decrease in AA-enriched phospholipids PC (16:0/20:4) (log_2_FC = -1.26) and PE (18:0/20:4) (log_2_FC = -0.82), suggesting that PLA_2_ activation drives AA to generate PGE_2_ via the COX pathway, constituting the lipid basis of neuroinflammatory disease (Lingaraju et al., 2021; Ko et al., 2026), and is independently associated with elevated peripheral IL-6 and TNF-α (Jones et al., 2022). After YWKS intervention, PC (16:0/20:4) was significantly reversed (log_2_FC_drug = +1.45), and linoleic acid metabolism, arachidonic acid metabolism, and endocannabinoid retrograde signals were simultaneously enriched (all 11 or 10 hits, *p* < 0.01), suggesting that multiple targets synergistically inhibit the AA inflammatory cascade.

Angiotensin I/II (5–8) levels increased in the SDP group (log_2_FC = +0.91), while the reversal was greatest after YWKS intervention (log_2_FC = -1.29). Repeated awakenings lead to increased renin secretion, which in turn promotes the accumulation of angiotensin II and the release of aldosterone. The brain’s RAS is also enhanced by AT_1_ receptors, creating a positive feedback loop that strengthens sleep disorders and RAS activation (Takeda et al., 2024; Shimoura et al., 2020; Xu et al., 2026). The simultaneous enrichment of renin-angiotensin, HIF-1, and insulin secretion pathways in KEGG reveals the systemic extension of metabolic disorders to the cardiovascular and endocrine systems. The simultaneous increase of estriol (log_2_FC = +1.36) and allotetrahydrocorticosterone (log_2_FC = +1.47) suggests the continuous activation of the HPA axis (Buckley and Schatzberg, 2005). The latter was effectively reversed by YWKS (log_2_FC = −1.07), suggesting that it partially restored the rhythmic regulation function of the HPA axis.

The Spearman correlation network identified 1137 significant metabolite pairs (|r|≥0.7, *p* < 0.05), and the Louvain algorithm divided them into 89 functional modules. The hub metabolites (connectivity ≥ 30) were concentrated in heterocyclic compounds and benzene ring derivatives. The top-ranked pivotal drugs are all exogenous. Their pivotal status may be due to combination therapy or cross-interference in MS/MS spectra, suggesting that metabolomics studies involving drug-treated subjects must establish a drug confounding effect correction process. L-serine was significantly downregulated in the SDP group (log_2_FC = -0.40), which was reversed after YWKS intervention (log_2_FC = +0.37). As a dual precursor to phosphatidylserine synthesis and D-serine production, its changes link amino acid metabolism disorders with neuroinflammation and synaptic plasticity damage (Holeček, 2022; Ye et al., 2021). The seven-part response model showed that YWKS reversed or normalized 68.8% (119/173) of disease-related DAMs, while 31.2% (54) showed no improvement; long-chain acylcarnitine and other mitochondrial dysfunction markers were particularly prominent, suggesting that mitochondrial-targeted adjuvant therapy has important translational value (Castillo-Vazquez et al., 2025; Lin et al., 2025).

In summary, this study systematically elucidated the panoramic disorder characteristics of plasma metabolomics in patients with sleep disorders and the metabolic remodeling trajectory of YWKS intervention through a multidimensional metabolomics framework, providing new systematic evidence for understanding the molecular pathological mechanisms of sleep disorders and the multi-target action mode of traditional drugs. Future research should focus on the synergistic effects of combining mitochondrial targeting strategies (such as coenzyme Q10, NAD^+^ precursors, etc.) with YWKS, in order to achieve more comprehensive therapeutic coverage of sleep disorders at the metabolic level.

## 5 Limitations of the study

This study is an exploratory pilot study, with a sample size in each group (n = 7) comparable to that of pilot studies in the same field of metabolomics. The study employed rigorous quality control procedures, OPLS-DA permutation tests, and multiple screening criteria to ensure data reliability as much as possible. Nevertheless, the existing sample size still has objective limitations in terms of statistical power and extrapolation of conclusions. Specifically, small sample sizes may reduce the detection sensitivity for low-abundance metabolites, increasing the risk of these metabolites being missed, thus leading to incomplete identification of potential biomarkers. Therefore, the results of this study should be interpreted within an exploratory framework. The identified metabolic biomarkers and related pathways provide a clear hypothetical framework for subsequent research and need to be further validated in larger-scale independent cohorts. In particular, multicenter studies are recommended to improve the generalizability and reproducibility of the conclusions.

Outlying drug metabolites represent a persistent limitation in cohorts receiving treatment; while identified compounds were omitted from the mechanistic discussion (Section 2.3), their contributions are difficult to separate from disease-related changes. Subsequent investigations should use medication-free participants, designs without simultaneous drug use, or stable-isotope labeling combined with stratified analysis to distinguish drug-induced signals from natural ones.

The mechanism explanation proposed in this paper is based solely on plasma metabolomics profiling and should only be considered as a research hypothesis. Further research could incorporate transcriptomics techniques (such as RT-PCR or RNA-seq to quantitatively detect key regulatory genes in purine metabolism and the mitochondrial β-oxidation pathway) to directly validate the mechanisms underlying the associations discovered in this study.

## 6 Conclusion

This study systematically identified five core metabolic disorder axes of sleep disorders in 352 plasma metabolites using non-targeted LC-MS/MS metabolomics: purine degradation-oxidative stress, tryptophan-serotonin circuit dysregulation, impaired mitochondrial fatty acid β-oxidation, neuroinflammation mediated by glycerophospholipid remodeling, and activation of the renin-angiotensin system, providing metabolomic evidence for multi-system metabolic dysregulation in sleep disorders as a basis for future mechanistic investigation. YWKS treatment was associated with normalization of 68.8% (119/173) of disease-related differential metabolites, with notable co-modulation of metabolites in the purine-XO axis and RAS pathway. However, long-chain acylcarnitine, represented by stearoylcarnitine, continued to increase (31.2%, 54 metabolites), suggesting that the mitochondrial β-oxidation function defect was not adequately corrected after YWKS treatment. The above findings provide systematic metabolomics evidence for the molecular pathological mechanisms of sleep disorders and suggest that future mitochondrial-targeted assistance strategies, such as coenzyme Q10 and NAD^+^precursors, may achieve a more comprehensive recovery of metabolic function. It is important to emphasize that these results are correlational, coming from a preliminary study with n = 7 in each group. Before any definitive mechanistic or treatment-related claims can be made, verification through functional assays and larger-scale controlled research is essential.

## Data Availability

The original contributions presented in the study are included in the article/Supplementary Material, further inquiries can be directed to the corresponding authors.
