# Supplementary material for: Untargeted plasma metabolomics reveals systemic metabolic dysregulation and reatment-associated metabolic modulation following YWKS treatment in sleep disorder patients
Source: Front Pharmacol. 2026 Sep 11;17:1905102. doi: 10.3389/fphar.2026.1905102 (PMC13612529; doi:10.3389/fphar.2026.1905102)
Supplement: Supplementary file 1 [file Supplementaryfile1.docx]

**Supplementary Materials**

Supplementary Methods: Diagnostic criteria, inclusion/exclusion criteria, intervention, and sampling time points

**Diagnostic criteria ( ICSD-3)**
The diagnosis of sleep disorders was established using the International Classification of Sleep Disorders, Third Edition (ICSD-3). Individuals were included if they met all criteria, including (1) chronic dissatisfaction with sleep amount or quality, as shown by trouble falling asleep, staying asleep, waking up early, or having non-restorative sleep at least three times weekly.(2) notable daytime suffering or impaired functioning; (3) persistent symptoms for at least three months; (4) sufficient sleep opportunities; and (5) the absence of alternative explanations such as other sleep disturbances, drug use, external conditions, or additional health issues.
**Inclusion criteria**
Individuals were included if they met the following benchmarks: (1) dual diagnostic compliance with Western and Uyghur medical criteria; (2) age 30–75 years; (3) voluntary involvement with written consent; (4) a score of 7–18 on the Pittsburgh Sleep Quality Index (PSQI); (5) mild or moderate insomnia; (6) a 1-to-6-month history of the condition; and (7) the consciousness and ability to engage with treatment and assessment forms.
**Exclusion criteria**
Criteria for exclusion included: (1) pregnancy or breastfeeding; (2) systemic conditions including pain, fever, or being in a perioperative state; (3) insomnia primarily attributed to environmental disturbances; and (4) serious concurrent illnesses affecting the cardiovascular, neurological, respiratory, endocrine, or hematopoietic systems, among other major somatic diseases.(5) psychological issues such as anxiety and depression; (6) an allergic diathesis or a documented reaction to the experimental treatment; (7) active use of substances influencing metabolism or sleep (for instance, stimulants and antidepressants); (8) lack of protocol adherence, missing information, participant attrition, or self-initiated withdrawal.

**Intervention**
Individuals in the SDPM group took 30 mL of Yinao Wusiti Kudusi Syrup (YNWS) by mouth twice daily over a 14-day period.
**Blood sampling time points**
Prior to sampling, initial data were gathered and necessary precautions were detailed. Following the signing of informed consent and associated paperwork, the baseline blood sample was obtained. Subjects then underwent treatment based on their group allocation, with a final blood draw conducted upon completion of the therapy course.
**Ethics**
The research protocol has been approved by the Ethics Committee of Xinjiang Uygur Autonomous Region Uygur Medicine Hospital (Second People's Hospital of Xinjiang Uygur Autonomous Region) (Approval No.: 2025-KY-20-03), and written informed consent has been obtained from all participants.

**Table 1 Baseline Clinical Characteristics**

| **Variable** | **Control (n=7)** | **SDP (n=7)** | **SDPM (n=7)** |
| --- | --- | --- | --- |
| Age, years (mean +/- SD) | 47.7 +/- 15.9 | 54.1 +/- 17.2 | 56.9 +/- 4.8 |
| Age range | 21-62 | 23-78 | 49-63 |
| Sex (M/F) | 3/4 | 3/4 | 5/2 |
| Disease duration, months (mean +/- SD) | N/A | 3.2 +/- 2.1 | 3.7 +/- 2.9 |
| Comorbidities | None | Coronary heart disease (n=1); Type 2 diabetes + coronary atherosclerosis (n=1) | Type 2 diabetes (n=2); Cerebral infarction (n=2); Cerebral hemorrhage (n=3); Central facial paralysis (n=1); Right hemiplegia (n=1) |

**Table 2. OPLS-DA model quality metrics for the three pairwise comparisons. All Q² values > 0.4 indicate robust, nonoverfitted models.**

| **Comparison** | **R²X** | **R²Y** | **Q²** |
| --- | --- | --- | --- |
| SDP vs Control | 0.236 | 0.996 | 0.578 |
| SDPM vs Control | 0.277 | 0.996 | 0.680 |
| SDPM vs SDP | 0.232 | 0.996 | 0.480 |

**Table 3. Seven-category response pattern classification of all 352 detected metabolites. Drug-reversible metabolites (categories 1–4): 119 (34%). Persistently altered (categories 5–6): 54 (15%).**

| **Response Pattern** | **Count** | **Biological Interpretation** |
| --- | --- | --- |
| Drug-reversed (SDP↑ → Drug↓) | 22 | Directly pharmacologically reversible upregulation |
| Drug-reversed (SDP↓ → Drug↑) | 9 | Directly pharmacologically reversible downregulation |
| Drug-normalized (SDP↑) | 68 | Elevated in SDP, restored to control levels by drug |
| Drug-normalized (SDP↓) | 20 | Reduced in SDP, restored to control levels by drug |
| Persistently elevated | 31 | Drug-resistant sleep disorder biomarker candidates |
| Persistently reduced | 23 | Persistent deficit despite treatment |
| Drug-specific change | 133 | Pharmacological effects not directly related to disease |

**Table 4, the enrichment of KEGG pathways is shown for three sets of pairwise comparisons.**

| **KEGG Pathway** | **Hits/Total** | **P-value** | **Comparison** |
| --- | --- | --- | --- |
| Glycerophospholipid metabolism | 9/81 | < 0.05 | SDP vs Control |
| Cysteine and methionine metabolism | 5/18 | < 0.05 | SDP vs Control |
| D-Amino acid metabolism | 5/30 | < 0.05 | SDP vs Control |
| ABC transporters | 6/— | < 0.05 | SDP vs Control |
| Biosynthesis of cofactors | 6/— | < 0.05 | SDP vs Control |
| Linoleic acid metabolism | 11/— | < 0.01 | SDPM vs Control |
| Arachidonic acid metabolism | 11/— | < 0.01 | SDPM vs Control |
| Retrograde endocannabinoid signaling | 10/— | < 0.01 | SDPM vs Control |
| Glycerophospholipid metabolism | 7/81 | < 0.05 | SDPM vs SDP |

**Table 5. Key differentially abundant metabolites across the mechanistic axes. Pipecolic acid, L-Serine, and Nicotinic acid were top-ranked by OPLS-DA VIP score.**

| **Metabolite** | **Class** | **SDP vs C (log₂FC)** | **SDPM vs SDP (log₂FC)** | **Key Finding** |
| --- | --- | --- | --- | --- |
| Hypoxanthine | Purine | +1.96 | −2.14 | Strongest drug reversal; xanthine oxidase activation |
| Stearoylcarnitine | FA/Acylcarnitine | +2.16 | +2.64 (vs C) | Persistent elevation; therapeutic gap |
| 5-Hydroxyindole | Tryptophan | +1.58 | −0.62 | 5-HT catabolism; partial drug reversal |
| Angiotensin I/II (5-8) | Peptide/Hormone | +0.91 | −1.29 | RAS activation; strong drug reversal |
| PC(16:0/20:4) | GP | −1.26 | +1.45 | AA-containing PL; drug restored |
| Estriol | Hormone | +1.36 | — | HPA axis activation |
| Allotetrahydrocorticosterone | Hormone | +1.47 | — | Adrenal steroid dysregulation |
| Nervonic acid | FA | +0.39 | Persistent | Myelin-related lipid; persistent |
| Allopurinol | Purine | +1.62 | −1.86 | XO inhibitor; paradoxical co-elevation |
| L-Serine | Amino acid | −0.40 | +0.37 | Neurotransmitter/phospholipid precursor |
| Allotetrahydrocorticosterone | Steroid | +1.47 | −1.07 | Adrenal steroid; HPA axis activation |

# Supplementary Figure S1. OPLS-DA Permutation Tests

Permutation tests (n=200) for all three pairwise comparisons. Purple bars: distribution of permuted Q2 values; orange bars: distribution of permuted R2Y values. Arrows indicate the actual model values. All Q2 permutation p-values < 0.05, confirming model robustness.

Supplementary Figure S1A. SDP vs Control: Q2 = 0.578 (p < 0.005, 0/200); R2Y = 0.996 (p = 0.27, 54/200)

Supplementary Figure S1B. SDPM vs Control: Q2 = 0.680 (p < 0.005, 0/200); R2Y = 0.996 (p = 0.015, 3/200)

Supplementary Figure S1C. SDPM vs SDP: Q2 = 0.480 (p = 0.03, 6/200); R2Y = 0.996 (p = 0.015, 3/200)
